# Determining folding and binding properties of the C‐terminal SH2 domain of SHP2

**DOI:** 10.1002/pro.4201

**Published:** 2021-10-09

**Authors:** Caterina Nardella, Francesca Malagrinò, Livia Pagano, Serena Rinaldo, Stefano Gianni, Angelo Toto

**Affiliations:** ^1^ Istituto Pasteur—Fondazione Cenci Bolognetti, Dipartimento di Scienze Biochimiche “A. Rossi Fanelli” and Istituto di Biologia e Patologia Molecolari del CNR Sapienza Università di Roma Rome Italy

**Keywords:** Chevron plot, Gab2, intermediate, kinetics, mutagenesis

## Abstract

SH2 domains are a class of protein–protein interaction modules with the function to recognize and bind sequences characterized by the presence of a phosphorylated tyrosine. SHP2 is a protein phosphatase involved in the Ras‐ERK1/2 signaling pathway that possess two SH2 domains, namely, N‐SH2 and C‐SH2, that mediate the interaction of SHP2 with various partners and determine the regulation of its catalytic activity. One of the main interactors of the SH2 domains of SHP2 is Gab2, a scaffolding protein with critical role in determining cell differentiation. Despite their key biological role and the importance of a correct native fold to ensure it, the mechanism of binding of SH2 domains with their ligands and the determinants of their stability have been poorly characterized. In this article, we present a comprehensive kinetic study of the folding of the C‐SH2 domain and the binding mechanism with a peptide mimicking a region of Gab2. Our data, obtained at different pH and ionic strength conditions and supported by site‐directed mutagenesis, highlight the role of electrostatic interactions in the early events of recognition. Interestingly, our results suggest a key role of a highly conserved histidine residue among SH2 family in the interaction with negative charges carried by the phosphotyrosine of Gab2. Moreover, the analysis of the equilibrium and kinetic folding data of C‐SH2 describes a complex mechanism implying a change in rate‐limiting step at high denaturant concentrations. Our data are discussed under the light of previous works on N‐SH2 domain of SHP2 and other SH2 domains.

## 1 INTRODUCTION

SH2 domains represent a class of protein–protein interaction domains with a highly conserved three‐dimensional structure within the proteome, characterized by 4–6 beta strands flanked by two alpha helices. They are able to recognize specific sequences characterized by the presence of a phosphorylated tyrosine. Despite tyrosine phosphorylation accounts just for ~0.5% of the total post‐translational modifications occurring in the eukaryotic cell, it has a fundamental role in regulating key molecular pathways. Because of this characteristic, SH2 domains are commonly found in proteins involved in signal transduction, making them important players on the field of cell physiology regulation. In fact, mutations causing an alteration of the interactions mediated by these domains are at the basis of many pathologies, including cancer.^1^, ^2^, ^3^, ^4^

SHP2 is a large phosphatase encoded by PTPN11 gene, with pivotal role in regulating several physiological aspects of the cell, as cell cycle control, differentiation, and migration,^5^ and in controlling oncogenic molecular pathways such as Jak/STAT,^6^, ^7^ PI3K/AKT,^8^, ^9^ and RAS/Raf/MAPK,^10^, ^11^, ^12^ thus representing an attractive target for cancer therapies. SHP2 mutations have been correlated with the onset of tumors like myelodysplastic syndrome and juvenile acute myeloid leukemia, melanoma, neuroblastoma, and colon cancer (listed in COSMIC database https://cancer.sanger.ac.uk/cosmic), as well as with syndromes, such as NOONAN and LEOPARD syndromes^13^, ^14^, ^15^ characterized by an increased propensity to develop cancer.

From a structural perspective SHP2 is composed by two SH2 domains (namely, N‐SH2 and C‐SH2 domains) followed by a PTP domain, that retains the catalytical activity of the protein. The two SH2 domains mediate the interaction of SHP2 mainly with scaffolding proteins. These interactions trigger the activation of the PTP domain inducing a major conformational change that releases the catalytical active site from the autoinhibition mediated by the N‐SH2 domain.^16^ The C‐SH2 domain on the other hand, by binding a second phosphotyrosine site of the ligand, recruits coordinates and orientates the binding partner increasing its concentration in the proximity of the protein, thermodynamically favoring the propensity of the autoinhibiting N‐SH2 domain to change its conformation.^16^

One of the main interactors of SHP2 in the cellular environment is Gab2, a scaffolding protein that serves as an important piece in the complicated puzzle of the assembling of several signaling systems.^17^, ^18^, ^19^ Apart from a structured Pleckstrin Homology domain (PH), Gab2 is characterized by a long disordered tail presenting several docking sites for adaptor proteins, such as SHP2, Grb2, p85, PLC‐g, CRK, SHC, and SHIP.^19^, ^20^ These interactions are finely regulated both temporally and spatially, to ensure a correct signal transduction. In particular, the binding of the SH2 domain of SHP2 with specific regions of Gab2 is at the basis of several molecular pathways,^20^ and mutations occurring on both proteins are reported as causative of a number of different tumor diseases, such as breast, lung and gastric cancer, leukemia and melanoma.^20^, ^21^, ^22^, ^23^

Characterizing the folding mechanism and the determinants of the stability of SH2 domains is a fundamental step towards understanding the molecular basis of their biochemical function and, consequently, of their role in the physiological pathways in which they are involved. In this article, we propose a comprehensive rigorous analysis of the folding pathway and binding mechanism of the C‐SH2 domain of SHP2 with a peptide mimicking a specific region of Gab2. From a folding perspective, the analysis of equilibrium and kinetic (un)folding data highlighted a three‐state folding mechanism implying the presence of a high‐energy metastable intermediate. On the other hand, by employing equilibrium and kinetic experiments conducted in a wide range of experimental conditions, changing pH and ionic strength of the solutions, and integrating those data with site‐directed mutagenesis, we provided a complete characterization of the mechanism of recognition and binding between C‐SH2 domain and Gab2. Our data are discussed in comparison with those obtained for the N‐SH2 domain of SHP2 and, more in general, under the light of previous works on SH2 domains.

## 2 RESULTS

### 2.1 Equilibrium unfolding experiments

**FIGURE 1:**
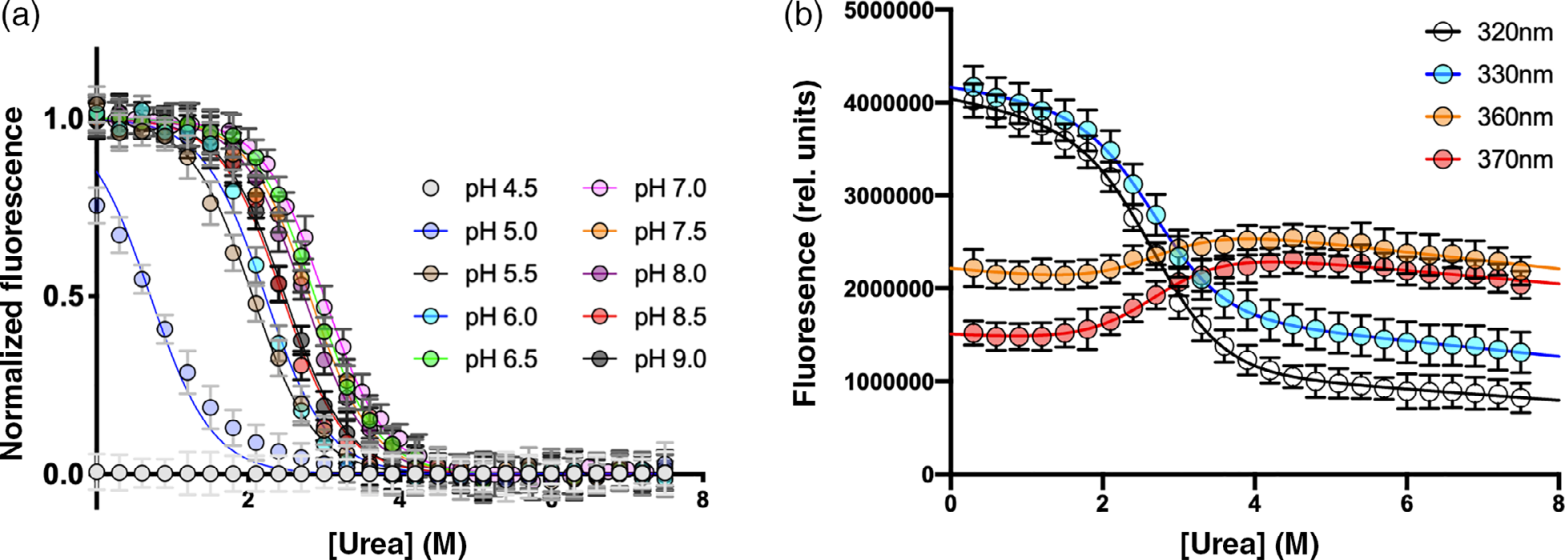
Panel (a): Equilibrium unfolding profiles of the C‐SH2 domain at different pH conditions. The dependence of the normalized fluorescence recorded at 330 nm as a function of the concentration of denaturant is reported. Panel (b): Urea‐induced denaturation performed at pH 8.0 and recorded at different wavelengths. In all cases data were globally fitted with a two state model equation, sharing the *m*_D–N_ value for all datasets (see details in the text)

The equilibrium unfolding of the His‐tagged C‐SH2 domain (only C‐SH2 from now on) was explored monitoring the intrinsic fluorescence of the tryptophane residue in position 112. The normalized fluorescence collected at 330 nm of urea induced equilibrium unfolding conducted in different pH conditions, at 298 K, are reported in Figure 1a. The reducing agent 1,4‐dithiothreitol (DTT) was added to the buffer at the concentration of 2 mM to reduce cysteine residues C104 and C174, which are not reported to covalently interact in the full length SHP2 protein (PDB: 2shp). The denaturation process showed a simple sigmoidal profile that is consistent with a two‐state folding mechanism, suggesting the absence of intermediates accumulating during the transition. Curves were globally fitted by sharing the *m*_D–N_ value, defined as δΔG/δ[denaturant], that is an index of the cooperativity of the reaction and it is correlated with the change of the accessible surface area upon unfolding.^24^ The *m*_D–N_ value calculated was 1.4 ± 0.1 kcal mol^−1^ M^−1^, consistent with a protein of 123 residues.^24^ Denaturation curves at different wavelengths recorded at pH 8.0 (Figure 1b) were satisfactorily fitted by sharing m_D‐N_ value and midpoint, supporting the hypothesis of absence of folding intermediate along the reaction pathway.

### 2.2 Kinetic (un)folding experiments

**FIGURE 2:**
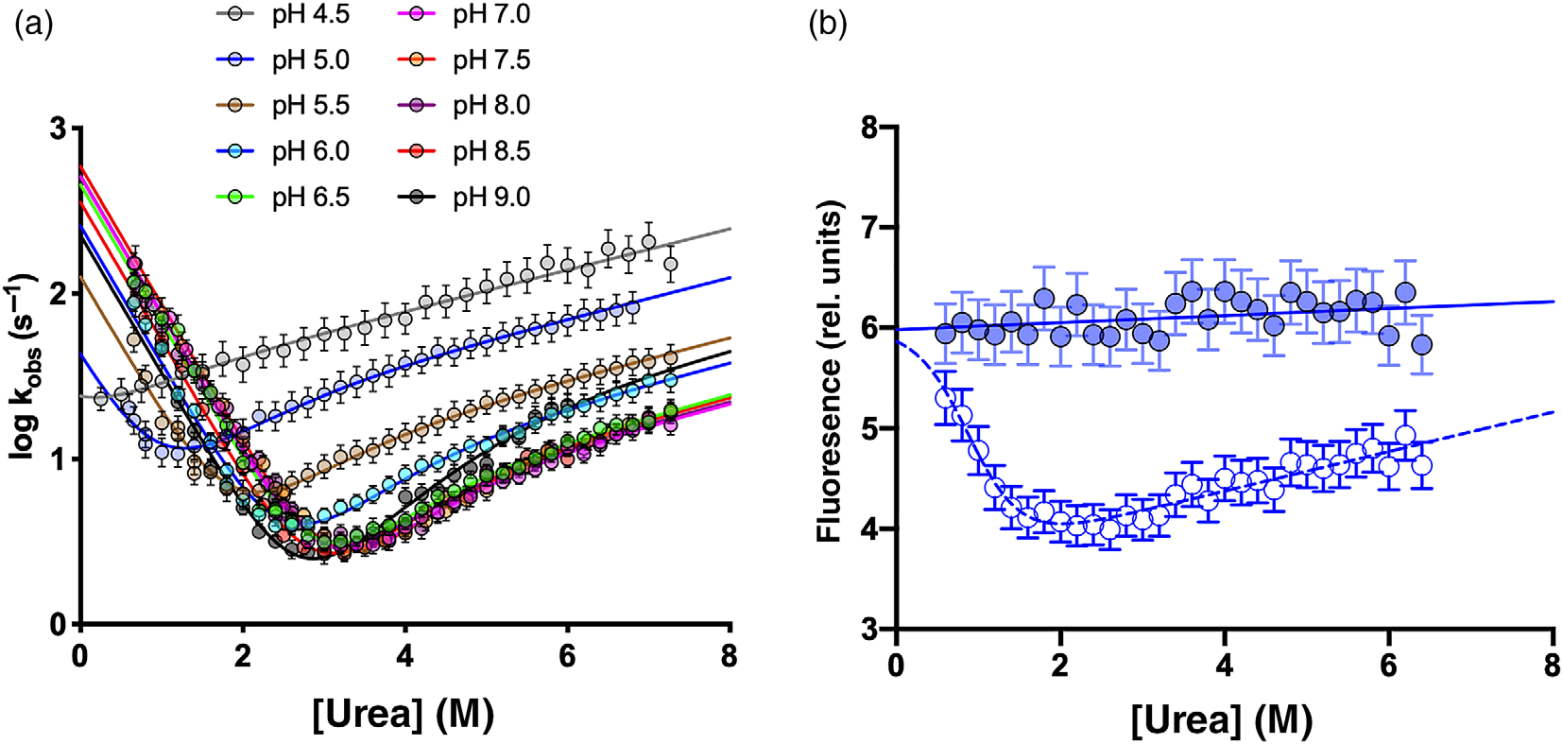
Panel (a): Dependence of the logarithm of the observed rate constants for unfolding and refolding experiments as a function of [urea] at different pH conditions. Lines are the best fit to an equation describing a change in rate‐limiting step at high [urea] with the presence of a high‐energy intermediate along the reaction pathway. Panel (b): Analysis of observed initial (filled circles) and final (empty circles) fluorescence signal recorded in kinetic unfolding experiments at pH 5.5. The initial fluorescence, which resembles the fluorescence emission of the native state, show a linear dependence as function of [urea], indicating the absence of burst‐phase events

Folding and unfolding kinetics of C‐SH2 domain were investigated at different pH conditions ranging from 4.5 to 9.0. At all the conditions explored, both folding and unfolding time courses were satisfactorily fitted with a single exponential equation. The logarithm of observed rate constants (*k*_obs_) were plotted versus the denaturant concentrations (chevron plots) at different pH conditions (Figure 2a). Interestingly, whilst the logarithm of the *k*_obs_ in the refolding arm appear to decrease linearly over denaturant concentrations, a deviation from linearity^25^ is clear in the unfolding arm of the chevron plots. This phenomenon, called “roll‐over effect,” is typically correlated with the presence of partially folded intermediate accumulating along the reaction pathway or with change in rate limiting step.^26^, ^27^, ^28^, ^29^, ^30^, ^31^ As shown above, analysis of thermodynamic parameters from equilibrium unfolding supports the hypothesis of the absence of low energy folding intermediate accumulating during the reaction. To further investigate this aspect, we analyzed the dependence of initial and final fluorescence signal of kinetic unfolding traces recorded at pH 5.5 at different denaturant concentrations (Figure 2b). It is clear that initial fluorescence signals, which resemble the fluorescence of native protein, display a simple linear dependence on urea concentration, indicating the absence of detectable burst‐phase unfolding events.^27^, ^29^ On these bases, chevron plots were fitted using the following equation

(1) $$k_{\mathrm{obs}}=k_{f}+\frac{k_{u}}{1+k_{\mathrm{IU}}/k_{\text{IN}}}$$

assuming the absence of populated low energy intermediate(s) and ascribing the curvature on the unfolding arm to a change in rate limiting step at different denaturant concentrations. The fitting process was performed by globally sharing kinetic *m*‐values (Table 1). The analysis of kinetic parameters allowed the calculation of the position of the two transition states, TS1 and TS2, along the reaction coordinate (Tanford *β* values), respectively, as *β*_TS1_ = 0.61 ± 0.03 and *β*_TS2_ = 0.91 ± 0.04.

**TABLE 1:** Kinetic folding parameters of the C‐SH2 domain of SHP2 calculated at different pH conditions

pH	*k*_*f*_ (s^−1^)	*k*_*u*_ (s^−1^)	*K*_part_
4.5	*	*	*
5.0	41 ± 4	3.10 ± 0.50	0.24 ± 0.04
5.5	126 ± 6	0.54 ± 0.11	0.10 ± 0.02
6.0	260 ± 10	0.14 ± 0.03	0.04 ± 0.01
6.5	460 ± 20	0.06 ± 0.02	0.03 ± 0.01
7.0	510 ± 20	0.05 ± 0.02	0.02 ± 0.01
7.5	590 ± 30	0.04 ± 0.01	0.02 ± 0.01
8.0	510 ± 20	0.06 ± 0.02	0.03 ± 0.01
8.5	360 ± 20	0.05 ± 0.02	0.02 ± 0.01
9.0	230 ± 10	0.06 ± 0.02	0.01 ± 0.01

*Note*: *m*‐values were obtained from global fitting of the entire data set at different pH, *m*_*f*_ = 1.14 ± 0.01 kcal mol^−1^ M^−1^, *m*_*u*_ = 0.73 ± 0.04 kcal mol^−1^ M^−1^, *m*_part_ = 0.56 ± 0.06 kcal mol^−1^ M^−1^. *K*_part_ value corresponds to *k*_IU_/*k*_IN_ as described in the text.

*Kinetic parameters at pH 4.5 could not be measured due to a destabilization of the protein.

### 2.3 Kinetic binding experiments

To investigate the mechanism of the binding reaction between the C‐SH2 domain and Gab2 we performed kinetic binding experiments using a stopped flow apparatus, by rapidly mixing the C‐SH2 domain with a peptide mimicking the region of Gab2 comprised from residue 637 to 649 (Gab2_637–649_ from now on), modified with a dansyl group covalently linked at the N‐terminus. This modification allowed us to monitor the binding reaction by Förster Resonance Energy Transfer (FRET) using the naturally present tryptophan residue in position 112 as donor and dansyl group as acceptor. Since SH2 domains recognize sequences characterized by the presence of a phosphorilated tyrosine, our aim was to address the role of electrostatic charges in the binding reaction between Gab2 and the C‐SH2 domain performing kinetic binding experiments at different ionic strengths and different pH conditions.

**FIGURE 3:**
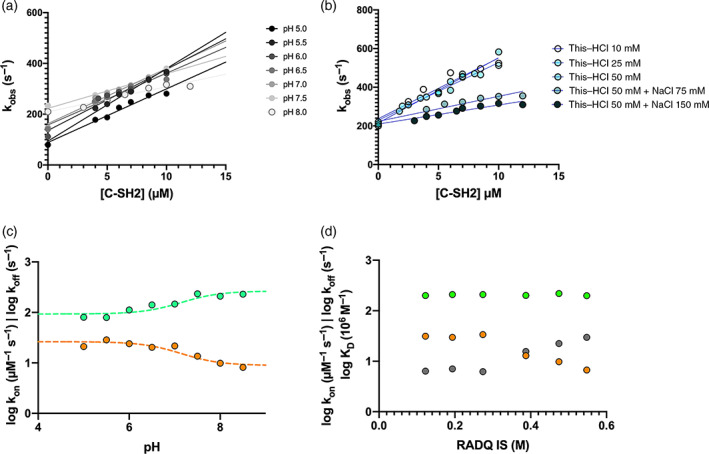
Panel (a): Pseudo‐first order kinetics of the binding reaction between dansyl‐Gab2 versus different concentrations of C‐SH2, at different pH conditions. As described in the text, points at 0 concentration of ligand were measured in separated displacement experiments. Panel (b): Pseudo‐first order kinetics of the binding reaction between dansyl‐Gab2 versus different concentrations of C‐SH2, at different ionic strength conditions (details of buffer used in Section 4 and in the legend). Panel (c): Dependence of logarithm of microscopic association (orange) and dissociation (green) rate constants as a function of pH. Lines are the best fit to Henderson–Hasselbalch equation. Panel (d): Dependence of logarithm of microscopic association (orange) and dissociation (green) rate constants and equilibrium dissociation rate constant (grey) as a function of ionic strength

The observed rate constants obtained by rapidly mixing a constant concentration of dansylated Gab2_637–649_ (2 μM) versus increasing concentrations of C‐SH2 domain (ranging from 2 to 14 μM) at 298 K, in different pH conditions (ranging from 5.0 to 8.0) and increasing ionic strength conditions at pH 8.0 (buffer Tris–HCl 10 mM, 25 mM, 50 mM, +75 mM, +150 mM NaCl at pH 8.0 in presence of 2 mM DTT; Figure 3a,b, respectively) were all fitted by a linear equation, the slope representing the microscopic association rate constant *k*_on_ of the reaction, and the intercept with *y*‐axis the microscopic dissociation rate constant *k*_off_. However, since a high experimental error may arise from the undirect measurement of the *k*_off_, we measured it directly performing a typical kinetic displacement experiment. A preincubated complex of dansyl‐Gab2_637‐649_ and C‐SH2 (both at the concentrations of 4 μM) was rapidly mixed with different concentrations of a competing reactant with different optical properties, that is, nondansylated Gab2_637–649_, in high excess (ranging from 40 μM to 80 μM). In agreement with theory,^32^ the observed rate constant calculated from displacement experiments were insensitive to displacer species concentrations. In all the experiments conducted, traces were satisfactorily fitted with a single‐exponential equation.

The dependence of the logarithm of microscopic association and dissociation rate constants, obtained at different pH conditions and ionic strengths are shown in Figure 3c,d (Table 2). Data revealed that, while the *k*_off_ remains unaffected as the ionic strength increases, there is a clear progressive decrease of the *k*_on_, from 31.4 ± 3.8 μM^−1^ s^−1^ at the lowest ionic strength to 6.4 ± 0.5 μM^−1^ s^−1^ at the highest ionic strength explored, resulting in a gradual decrease of the affinity of the complex, confirming the electrostatic nature of the early recognition events.

**TABLE 2:** Kinetic parameters obtained from pseudo‐first order binding experiments at different pH conditions for wild‐type C‐SH2 domain and histidine‐to‐alanine variants

pH	WT			H114A		
	*k*_on_ (μM^−1^ s^−1^)	*k*_off_ (s^−1^)	*K*_*D*_ (μM)	*k*_on_ (μM^−1^ s^−1^)	*k*_off_ (s^−1^)	*K*_*D*_ (μM)
5.0	21.1 ± 0.5	80 ± 2	3.8 ± 0.2	10.8 ± 0.6	92 ± 17	8.5 ± 0.2
5.5	28.6 ± 0.6	79 ± 2	2.8 ± 0.1	32.0 ± 7.0	102 ± 3	3.2 ± 0.1
6.0	24.1 ± 0.8	112 ± 5	4.7 ± 0.1	52.0 ± 3.0	148 ± 7	2.9 ± 0.1
6.5	20.0 ± 1.0	141 ± 5	6.9 ± 0.1	24.0 ± 2.0	125 ± 14	5.2 ± 0.6
7.0	22.0 ± 2.0	147 ± 6	6.8 ± 0.2	31.0 ± 4.0	144 ± 5	4.7 ± 0.5
7.5	13.6 ± 0.9	234 ± 12	17.2 ± 0.3	21.0 ± 7.0	253 ± 26	12.0 ± 1.0
8.0	9.9 ± 0.4	210 ± 10	21.3 ± 0.4	23.0 ± 7.0	263 ± 26	12.0 ± 1.0

pH	H116A			H132A		
	*k*_on_ (μM^−1^ s^−1^)	*k*_off_ (s^−1^)	*K*_*D*_ (μM)	*k*_on_ (μM^−1^ s^−1^)	*k*_off_ (s^−1^)	*K*_*D*_ (μM)
5.0	16.0 ± 2.0	103 ± 2	6.6 ± 0.1	22.0 ± 2.0	80 ± 2	3.6 ± 0.2
5.5	29.0 ± 5.0	98 ± 6	3.4 ± 0.2	29.0 ± 7.0	69 ± 1	2.4 ± 0.1
6.0	19.0 ± 3.0	105 ± 12	5.6 ± 0.1	45.0 ± 4.0	91 ± 2	2.0 ± 0.1
6.5	20.0 ± 2.0	130 ± 15	6.4 ± 0.2	14.0 ± 1.0	143 ± 10	10.4 ± 0.2
7.0	15.0 ± 1.0	170 ± 6	11.0 ± 1.0	20.0 ± 3.0	168 ± 7	8.5 ± 0.5
7.5	11.0 ± 5.0	204 ± 20	20.0 ± 2.0	18.0 ± 2.0	219 ± 15	12.0 ± 2.0
8.0	14.0 ± 3.0	196 ± 16	14.0 ± 1.0	11.0 ± 4.0	287 ± 29	27.0 ± 2.0

pH	H143A			H196A		
	*k*_on_ (μM^−1^ s^−1^)	*k*_off_ (s^−1^)	*K*_*D*_ (μM)	*k*_on_ (μM^−1^ s^−1^)	*k*_off_ (s^−1^)	*K*_*D*_ (μM)
5.0	11.0 ± 2.0	154 ± 5	14.3 ± 0.5	24.0 ± 1.0	125 ± 4	5.3 ± 0.1
5.5	32.0 ± 1.0	127 ± 5	4.0 ± 0.4	40.0 ± 3.0	114 ± 2	2.9 ± 0.2
6.0	20.0 ± 3.0	149 ± 10	7.4 ± 0.4	21.0 ± 4.0	116 ± 4	5.4 ± 0.4
6.5	9.0 ± 3.0	226 ± 23	25.0 ± 3.0	39.0 ± 2.0	132 ± 7	3.4 ± 0.1
7.0	8.0 ± 1.0	183 ± 12	23.0 ± 2.0	37.0 ± 2.0	125 ± 4	3.4 ± 0.2
7.5	13.0 ± 4.0	225 ± 27	17.0 ± 1.0	13.0 ± 1.0	172 ± 10	13.0 ± 1.0
8.0	14.0 ± 2.0	148 ± 19	10.8 ± 0.8	12.0 ± 2.0	162 ± 17	13.0 ± 2.0

**FIGURE 4:**
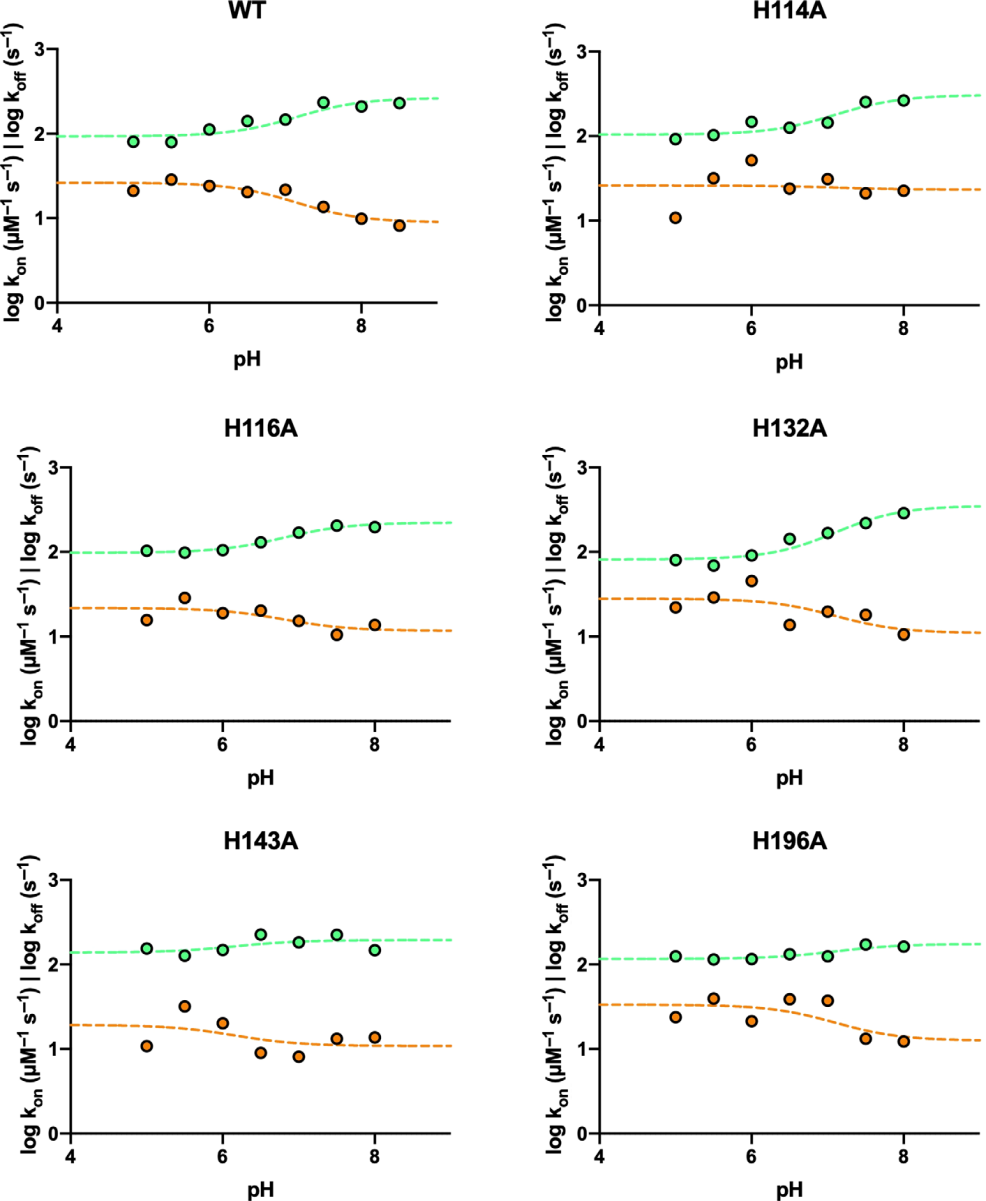
Dependence of logarithm of microscopic association (orange) and dissociation (green) rate constants as a function of pH for H114A, H116A, H132A, H143A, and H196A variants. Kinetic parameters for H169A variant could not be measured (details in the text). Lines are the best fit to Henderson–Hasselbalch equation

Additional insights came from the analysis of the dependence of the logarithm of *k*_on_ and *k*_off_ on pH, both displaying a clear sigmoidal behavior (Figure 3c). Data were consistent with the protonation of a single group with a pKa of 7.1 ± 0.2, a value close to the pKa of the lateral chain of histidine (6.04). Thus, to further investigate the role of histidine residue(s) in the binding reaction we resorted to mutate all histidines of the C‐SH2 domain into alanine, and monitor the effect of the mutation on binding kinetics. Variants H114A, H116A, H132A, H143A, H169A, and H196A were produced and employed in kinetic binding experiments conducted at different pH conditions (Table 2). Interestingly, the dependence of the logarithm of *k*_on_ and *k*_off_ obtained for the other variants were satisfactorily fitted with the same equation used for wt, sharing the pK_a_ value of 7.1 for all the data sets (Figure 4), whilst for H169A no binding traces could be recorded. To obtain additional information about this aspect, we resorted to investigate the binding of H169A mutant through an additional technique (Isothermal Titration Calorimetry). Calorimetric analysis of the binding of H169A mutant with Gab2_637–649_ showed indeed a remarkable change in affinity, suggesting that the missing kinetics were likely ascribable to an effect on the complex stability (Figure S1). Taken all together, our results suggest the protonation of residue H169 as responsible of the sigmoidal profile reported in Figure 3c.

## 3 DISCUSSION

Despite their abundance in the proteome, our knowledge about the folding mechanism of SH2 domains is very limited, with only few experimental data available.^33^, ^34^, ^35^, ^36^, ^37^, ^38^ Given their key role in cell physiology and their involvement in several human diseases, understanding the determinants of SH2 domain stability, strictly correlated with proper folding and accurate function in the recognition of specific ligands, appears a fundamental task to complete. To achieve this goal, a powerful methodology to determine the biophysical properties of a given protein system relies in its comparison with other proteins belonging to the same family, generally characterized by similar topology and function but with different primary structures.

**FIGURE 5:**
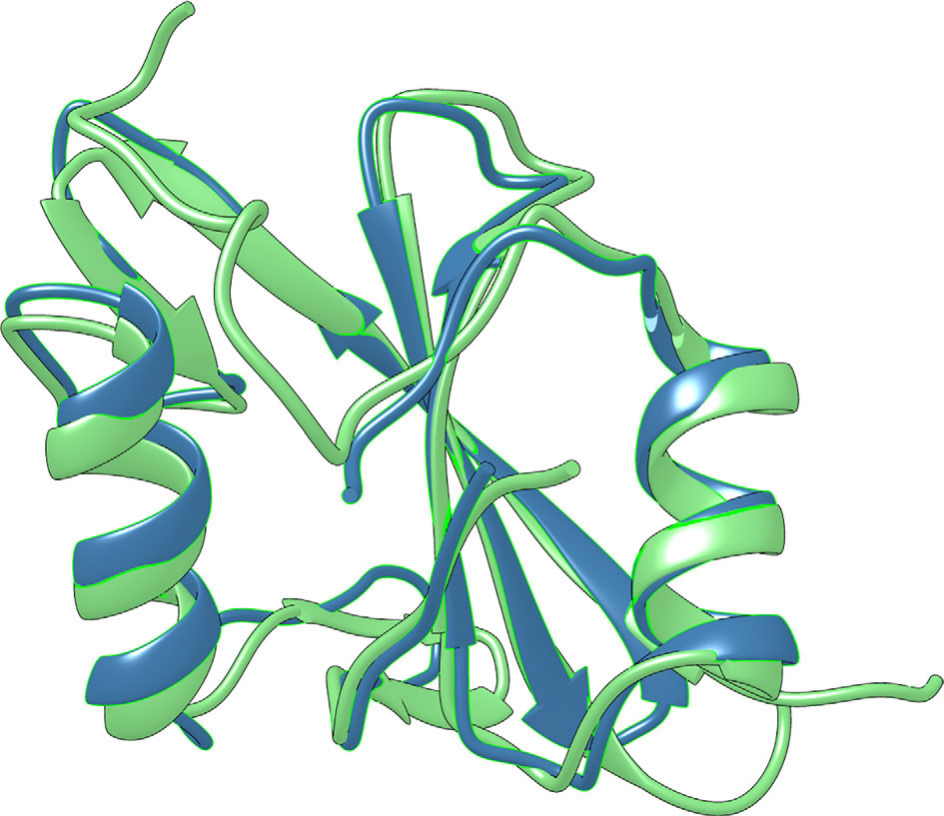
Comparison of the three‐dimensional structure of the N‐SH2 domain (in blue) and C‐SH2 domain (in green) of SHP2. Reported structured are from PDB: 4qsy and PDB:4jeg, respectively. Structural alignment and corresponding image were performed produced with the UCSF Chimera software

The SHP2 protein possesses a tandem of two SH2 domains, the N‐SH2 and the C‐SH2,^16^ with an almost superimposable three‐dimensional structure (Figure 5). The analysis of sequence identity performed with ClustalW server reported a score of 41.2%, highlighting a high sequence identity between the two domains. The folding pathway of the N‐SH2 domain was recently extensively characterized^33^, ^34^ and we resorted to compare it with the folding kinetic data obtained for the C‐SH2 domain. Kinetic (un)folding data were compatible with a three‐state folding mechanism, with the accumulation of a productive on‐pathway intermediate along the reaction. A structural characterization of the early and late folding steps was provided through Φ‐value analysis, highlighting a diffuse native‐like structure in the intermediate state, further locked in place by increasing native‐like contacts in the second transition state. On the other hand, the dependence of (un)folding kinetic observed rate constants over denaturant concentrations of the C‐SH2 domain revealed a curvature in the unfolding arm of the chevron plots at different pH conditions. Curvatures in chevron plots are typically interpreted as signature of the presence of intermediate(s) along the reaction pathway.^39^, ^40^, ^41^ However, the quantitative analysis of kinetic data may be performed with equations considering an intermediate accumulating along the reaction pathway or a change in rate limiting step. It is of interest to notice that the analysis of kinetic traces obtained in unfolding experiments performed at pH 5.5 revealed a linear dependence of the initial fluorescence over denaturant concentrations, with the absence of burst‐phase (Figure 2b). Our results may be also interpreted with a mechanism implying the presence of a low energy intermediate resembling the native state in its fluorescence properties. However, in contrast with what was previously observed for the N‐SH2 domain, C‐SH2 was found unable to bind 1‐anilinonaphtalene‐8‐sulphonate (ANS) (data not shown), a fluorescent dye that binds to hydrophobic clusters generally found in partially folded proteins. This result, together with data obtained from equilibrium unfolding experiments, suggests a mechanism in which the intermediate is a high‐energy species, never accumulating along the reaction. On the basis of the data of this work and of previous works on the folding of SH2 domains,^33^, ^34^, ^35^, ^36^, ^37^ our findings support the hypothesis that the presence of multiple energetic minima in the energy landscape of SH2 domains is not necessary for productive folding and may be only ascribable to an intrinsic propensity selected by the sequence of a single domain to form partially folded state. Future works will clarify the possible general validity of this mechanism.

SH2 domains exert the important biological function to recognize and bind specific sequences characterized by the presence of a phosphorylated tyrosine. In SHP2 protein the N‐SH2 and C‐SH2 domains work in tandem by binding two different sequences of the same substrate, the first by undergoing a conformational change and regulating the activity of the phosphatase domain, and the latter by orientating and increasing the concentration of the substrate in proximity of the protein.^16^ Importantly, SH2 domains of SHP2 show a high selectivity, the N‐SH2 domain being unable to recognize and bind Gab2_637–649_ in stopped‐flow kinetic binding experiments (Figure S2). Based on this evidence, it is not surprising the difference in affinity measured for the two domains in binding their specific recognized portions of Gab2. In particular, whilst for the N‐SH2 domain a nanomolar equilibrium dissociation rate constant was measured for Gab2 in the absence of additional salt in the experimental buffer,^33^ we here reported a ~1,000‐fold higher *K*_*D*_ in the binding of the C‐SH2 domain for Gab2 in comparable ionic strength conditions. This finding supports the scenario in which the C‐SH2 domain plays a secondary role in the binding of SHP2 substrates. The mechanism of recognition and binding of the substrate appears to be conserved between the two domains. Dependence of microscopic association and dissociation rate constant to the ionic strength of the solution clearly show an effect on *k*_on_, whilst the *k*_off_ remains almost insensitive to the concentration of NaCl. Thus, the early recognition event appears to be mostly driven by electrostatic charges carried by the phosphotyrosine and charged residues in the binding pocket, analogously to what has been previously reported for the N‐SH2 domain of SHP2.^33^

**FIGURE 6:**
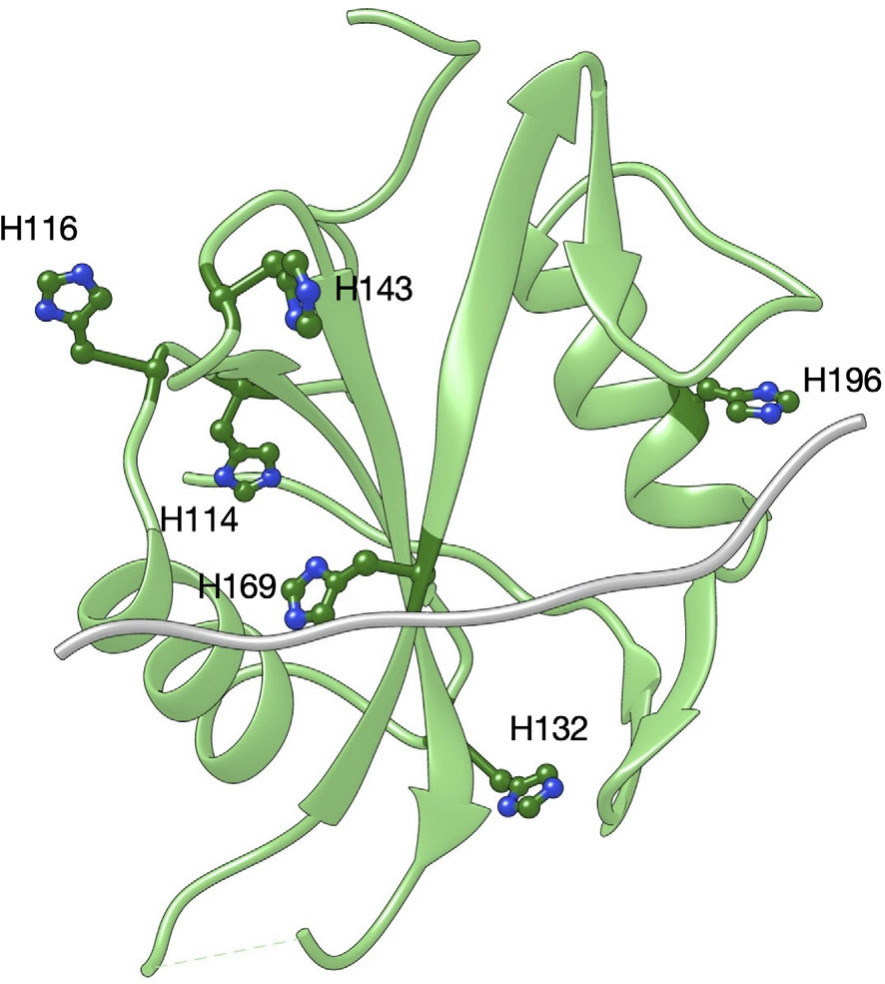
Three‐dimensional structure of the C‐SH2 domain from PDB:4jeg with H114, H116, H132, H143, H169, and H196 in dark green color and ball‐and‐sticks format. In grey, it is reported a general ligand in order to highlight the position H169 residue in the binding pocket of the C‐SH2 domain

The pH dependence of the binding reaction, together with a His‐to‐Ala mutational analysis of the binding kinetics highlights a pivotal role of residue H169 in determining the change in complex affinity measured at different pH values and in the recognition and binding events with Gab2. In fact, although H169A variation did not allow us to measure binding at the stopped‐flow apparatus, ITC experiments reported a remarkable effect on the affinity for Gab2 (Figure S1). Due to the position of H169 residue in the binding pocket (Figure 6), mutation into Alanine could determine an overall destabilization of the complex^42^, ^43^ and/or sub‐millisecond kinetics that could not be resolved by the stopped‐flow. In support to this hypothesis, H169 appears to be highly conserved among SH2 domains.^44^ Moreover, structural studies have been proposed H169 to be part of a complex hydrogen bonding network optimizing the recognition of the phosphate group covalently linked to the tyrosine residue of Gab2.^45^, ^46^ A combination of extensive mutagenesis and kinetic binding experiments will extend our understanding of the binding mechanism of the C‐SH2 domain of SHP2 and in general of SH2 domains.

## 4 MATERIALS AND METHODS

### 4.1 Protein expression and purification

The construct encoding the C‐SH2 domain of SHP2 protein (residues 97–220) was subcloned in a pET28b + plasmid vector and then transformed in Escherichia coli cells BL21 (DE3). Bacterial cells were grown in LB medium, containing 30 μg/ml of kanamycin, at 37°C until OD600 = 0.7–0.8, and then protein expression was induced with 0.5 mM IPTG. After induction, cells were grown at 25°C overnight and then collected by centrifugation. To purify the His‐tagged protein, the pellet was resuspended in buffer made of 50 mM Tris–HCl, 300 mM NaCl, Imidazole 10 mM, pH 8.0, with the addition of antiprotease tablet (Complete EDTA‐free, Roche), then sonicated and centrifuged. The soluble fraction from bacterial lysate was loaded onto a nickel‐charged HisTrap Chelating HP (GE Healthcare) column equilibrated with 50 mM Tris–HCl, 300 mM NaCl, Imidazole 10 mM, pH 8.0. Protein was then eluted with a gradient from 0 to 1 M imidazole by using an ÄKTA‐prime system. Fractions containing the protein were collected and the buffer was exchanged to 50 mM Tris–HCl, 150 mM NaCl, pH 8.0 by using a HiTrap Desalting column (GE Healthcare). The purity of the protein was analyzed through SDS‐page. Site‐directed mutagenesis was performed using the QuikChange mutagenesis kit (Agilent Technologies Inc., Santa Clara, CA), accordingly to manufacturer instructions. Peptides mimicking the region 637–649 of Gab2, with and without the dansyl N‐terminal modification, were purchased from GenScript.

### 4.2 Equilibrium experiments

Equilibrium unfolding experiments were performed on a Fluoromax single photon counting spectrofluorometer (Jobin‐Yvon, NJ). C‐SH2 domain was excited at 280 nm and emission spectra were recorded between 300 and 400 nm, at increasing urea concentrations. Experiments were performed with the protein at constant concentration of 2 μM, at 298 K, using a quartz cuvette with a path length of 1 cm. Buffers used for pH dependence were: 50 mM Acetate pH 4.5, 50 mM Acetate pH 5.0, 50 mM Acetate, pH 5.5, Bis–Tris 50 mM pH 6.0, Bis–Tris 50 mM pH 6.5, Hepes 50 mM pH 7.0, Hepes 50 mM pH 7.5, Tris–HCl 50 mM pH 8.0, Tris–HCl 50 mM pH 8.5, Tris–HCl 50 mM pH 9.0. Of note, 2 mM 1,4‐dithiothreitol (DTT) was added to all buffers. Data were fitted using Equation (1):

### 4.3 Stopped‐flow (un)folding experiments

Kinetic (un)folding experiments were performed on an Applied Photophysics Pi‐star 180 stopped‐flow apparatus, monitoring the change of fluorescence emission, exciting the sample at 280 nm and recording the fluorescence emission by using a 360 nm cutoff glass filter. The experiments were performed at 298 K, by using urea as denaturant agent. The buffers used were the same described in the Equilibrium Experiment paragraph. For each denaturant concentration, at least five individual traces were averaged. The final protein concentration was typically 2 μM.

### 4.4 ANS binding experiments

Unfolding and refolding kinetic experiments were performed on an Applied Photophysics Pi‐star 180 stopped‐flow apparatus. C‐SH2 at final concentration of 2 μM, in the presence of 50 mM Tris–HCl pH 8.0, 2 mM DTT, and 300 μM 1‐anilinonaphthalene‐8‐sulfonate (ANS), was excited at 280 nm and ANS fluorescence was recorded using a 455 nm cutoff filter.

Equilibrium experiments were performed on a Fluoromax single photon counting spectrofluorometer (Jobin‐Yvon, NJ), in absence and in the presence of different urea concentrations ranging from 0 to 7.5 M. Sample was excited at 350 nm and fluorescence emission was recorded in a quartz cuvette with 1 cm path length, between 400 and 600 nm. Final conditions were 2 μM C‐SH2, 50 mM Tris–HCl pH 8.0, 2 mM DTT, and 300 μM ANS at 298 K.

### 4.5 Stopped‐flow binding experiments

Kinetic binding experiments were performed on an Applied Photophysics sequential‐mixing DX‐17MV stopped‐flow apparatus (Applied Photophysics), set up in single mixing mode. Pseudo‐first order binding experiments were performed mixing a constant concentration (2 μM) of dansyl‐Gab2_637‐649_ with increasing [C‐SH2], from 2 to 14 μM. Samples were excited at 280 nm, and the emission fluorescence was recorded by using a 475 nm cutoff filter. Experiments were performed at 283 K. The buffers used for pH dependence were the same described above. For ionic strength dependence, buffers used were Tris–HCl 10 mM, Tris–HCl 25 mM, Tris–HCl 50 mM, Tris–HCl 50 mM NaCl 75 mM, Tris–HCl 50 mM NaCl 150 mM, pH 8.0. Of note, 2 mM DTT was added to all the buffers. For each acquisition, five traces were collected and averaged and satisfactorily fitted to a single exponential equation.

### 4.6 Stopped‐flow displacement experiments

As detailed in the text, microscopic dissociation rate constants were measured by performing displacement experiments on an Applied Photophysics sequential‐mixing DX‐17MV stopped‐flow apparatus (Applied Photophysics), set up in single mixing mode. A preincubated complex of C‐SH2 domain and dansyl‐Gab2_637‐649_ at constant concentration of 4 μM was rapidly mixed with different concentrations of an excess of non dansylated Gab2_637–649_, ranging from 40 μM to 820 μM. Samples were excited at 280 nm and fluorescence emission was collected by using a 475 nm cutoff filter. Experiments were performed at 283 K. The observed rate constants were calculated from the average of five single traces. Observed kinetics was consistent with a single exponential decay.

## AUTHOR CONTRIBUTIONS

**Caterina Nardella:** Formal analysis (equal); investigation (lead); methodology (equal). **Francesca Malagrinò:** Investigation (equal); methodology (equal). **Livia Pagano:** Investigation (equal); methodology (equal). **Serena Rinaldo:** Formal analysis (equal); methodology (equal). **Stefano Gianni:** Conceptualization (equal); resources (lead); supervision (equal); writing – review and editing (equal). **Angelo Toto:** Conceptualization (equal); supervision (equal); writing – original draft (lead); writing – review and editing (lead).

## Supporting information


**Figure S1** To analyze the specificity and the thermodynamics of the interaction, binding of Gab2_637–649_ peptide to either CSH2 wt. or H169A variant was directly measured using Isothermal Titration Calorimetry (ITC). Of note, 5 μM protein was titrated with 109 μM peptide solutions; the titration profile is depicted in the upper panel (black and green trace for the wt. and H169A mutant, respectively). Integration of the titration peaks leads to a sigmoidal curve (lower panel, black and green symbols for the wt. and H169A mutant, respectively), showing the heat evolved per mole of ligand injected versus the molar ratio of ligand to protein. The heat of binding (Δ*H*), the stoichiometry (*n*), and the dissociation constant (*K*_d_) were then calculated from this plot by fitting data with the “one‐binding‐site model” of the MicroCal version of ORIGIN (continuous lines). Experiments were carried out using an iTC200 microcalorimeter (MicroCal). Both protein and peptide solutions were buffer‐exchanged in buffer acetate 50 mM, 1 mM TCEP, pH 5.5. Twenty‐six injections of 1.5‐μl aliquots of peptide solution were injected into protein solution at 25°C, with a spacing of 120 s for each injection. Values are the means of two independent experiments ± *SD*.

**Figure S2** Comparison of the change in FRET signal obtained by rapidly mixing 2 μM of dansylated Gab2_637–649_ versus 10 μM of C‐SH2 domain (in black) and N‐SH2 domain (in gray), in buffer Hepes 50 mM, NaCl 150 mM, pH 7.0 at 283 K. It is clear that, while for the C‐SH2 domain the FRET occurring between the tryptophan donor and the dansyl acceptor is highly efficient, no change could be recorded for the N‐SH2 domain, due to the absence of binding reaction. This result demonstrates that Gab2_637–649_ is specifically recognized only by the C‐SH2 domain of SHP2.

**
**Table S1**** Supporting information
