# Supplementary figures and images for: Determining folding and binding properties of the C‐terminal SH2 domain of SHP2
Source: Protein Sci. 2021 Oct 9;30(12):2385–95. doi: 10.1002/pro.4201 (PMC8605372; doi:10.1002/pro.4201)

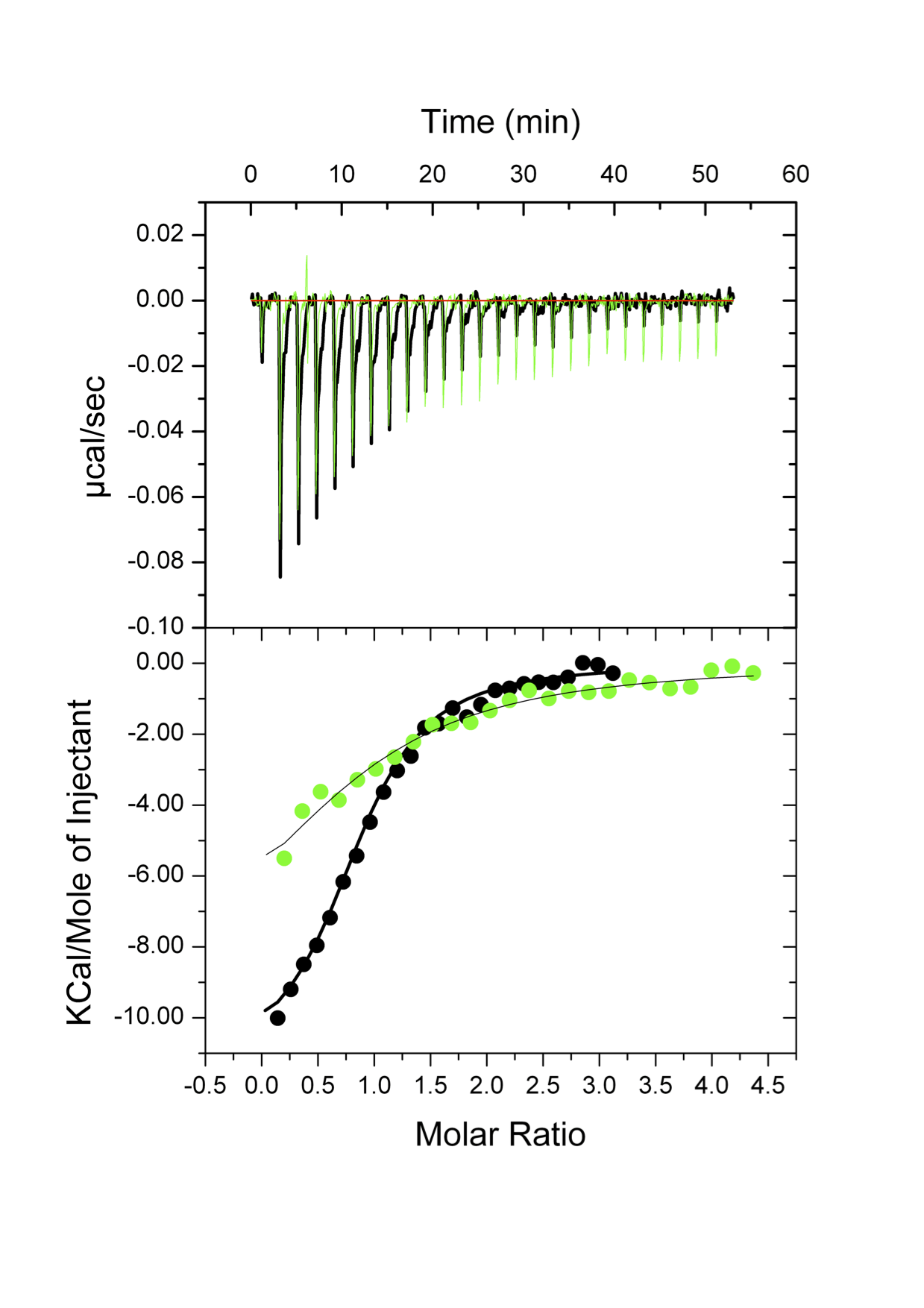

Supplement: Supplementary file 1 — Figure S1 To analyze the specificity and the thermodynamics of the interaction, binding of Gab2637–649 peptide to either CSH2 wt. or H169A variant was directly measured using Isothermal Titration Calorimetry (ITC). Of note, 5 μM protein was titrated with 109 μM peptide solutions; the titration profile is depicted in the upper panel (black and green trace for the wt. and H169A mutant, respectively). Integration of the titration peaks leads to a sigmoidal curve (lower panel, black and green symbols for the wt. and H169A mutant, respectively), showing the heat evolved per mole of ligand injected versus the molar ratio of ligand to protein. The heat of binding (ΔH), the stoichiometry (n), and the dissociation constant (K d) were then calculated from this plot by fitting data with the “one‐binding‐site model” of the MicroCal version of ORIGIN (continuous lines). Experiments were carried out using an iTC200 microcalorimeter (MicroCal). Both protein and peptide solutions were buffer‐exchanged in buffer acetate 50 mM, 1 mM TCEP, pH 5.5. Twenty‐six injections of 1.5‐μl aliquots of peptide solution were injected into protein solution at 25°C, with a spacing of 120 s for each injection. Values are the means of two independent experiments ± SD. [file PRO-30-2385-s003.TIF]

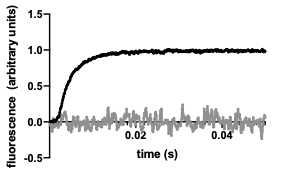

Supplement: Supplementary file 2 — Figure S2 Comparison of the change in FRET signal obtained by rapidly mixing 2 μM of dansylated Gab2637–649 versus 10 μM of C‐SH2 domain (in black) and N‐SH2 domain (in gray), in buffer Hepes 50 mM, NaCl 150 mM, pH 7.0 at 283 K. It is clear that, while for the C‐SH2 domain the FRET occurring between the tryptophan donor and the dansyl acceptor is highly efficient, no change could be recorded for the N‐SH2 domain, due to the absence of binding reaction. This result demonstrates that Gab2637–649 is specifically recognized only by the C‐SH2 domain of SHP2. [file PRO-30-2385-s001.tiff]
