# Supplementary material for: Determining folding and binding properties of the C‐terminal SH2 domain of SHP2
Source: Protein Sci. 2021 Oct 9;30(12):2385–95. doi: 10.1002/pro.4201 (PMC8605372; doi:10.1002/pro.4201)
Supplement: Supplementary file 3 — Table S1 Supporting information [file PRO-30-2385-s002.docx]

**Supplementary Material**

TABLE S1

| **Variant** | **n** | **K_a_ (M^-1^)** | **K_d_ (µM)** | **ΔH (kcal/mol)** | **ΔS (cal/mol/deg)** |
| --- | --- | --- | --- | --- | --- |
| WT | 0.7 ± 0.2 | (8.9 ± 1.1)⋅10^5^ | 1.1 ± 0.1 | -12.7 ± 1.8 | -15.6 ± 6.4 |
| H169A | 0.9 ± 0.1 | (2.4 ± 0.6)⋅10^5^ | 4.0 ± 0.1 | -9.7 ± 0.4 | -8.1 ± 1.4 |
